# Lipid Uptake, Metabolism, and Transport in the Larval Zebrafish

**DOI:** 10.3389/fendo.2017.00319

**Published:** 2017-11-20

**Authors:** Vanessa H. Quinlivan, Steven A. Farber

**Affiliations:** ^1^Carnegie Institution for Science (CIS), Baltimore, MD, United States; ^2^The Johns Hopkins University, Baltimore, MD, United States

**Keywords:** lipid metabolism, zebrafish, lipoproteins, comparative physiology, enterocytes

## Abstract

The developing zebrafish is a well-established model system for studies of energy metabolism, and is amenable to genetic, physiological, and biochemical approaches. For the first 5 days of life, nutrients are absorbed from its endogenous maternally deposited yolk. At 5 days post-fertilization, the yolk is exhausted and the larva has a functional digestive system including intestine, liver, gallbladder, pancreas, and intestinal microbiota. The transparency of the larval zebrafish, and the genetic and physiological similarity of its digestive system to that of mammals make it a promising system in which to address questions of energy homeostasis relevant to human health. For example, apolipoprotein expression and function is similar in zebrafish and mammals, and transgenic animals may be used to examine both the transport of lipid from yolk to body in the embryo, and the trafficking of dietary lipids in the larva. Additionally, despite the identification of many fatty acid and lipid transport proteins expressed by vertebrates, the cell biological processes that mediate the transport of dietary lipids from the intestinal lumen to the interior of enterocytes remain to be elucidated. Genetic tractability and amenability to live imaging and a range of biochemical methods make the larval zebrafish an ideal model in which to address open questions in the field of lipid transport, energy homeostasis, and nutrient metabolism.

## Introduction

The developing digestive system of the embryonic and larval zebrafish is a well-established model system for the study of vertebrate gastrointestinal physiology and metabolism. Metabolic and regulatory pathways for gastrointestinal system development, intestinal and liver cell differentiation, digestion, and nutrient uptake and transport are highly conserved between zebrafish and humans (1–7). The functional regionalization of the intestine also appears to be conserved among vertebrates including zebrafish with respect to transcription factor expression in epithelial cells over the length of the intestine (8). Additionally, the transparency of the developing larva makes it ideal for live imaging experiments: The larval zebrafish has a functional and visible liver, pancreas, gallbladder, intestine, and intestinal microbiota by 5 days post-fertilization (dpf) when it begins to feed. The zebrafish is also suitable for large-scale and high-throughput experiments due to its small size and high fertility (a single pair can produce hundreds of embryos in a day). Finally, as the importance of the gut microbiome to studies of nutritional physiology is becoming increasingly clear; the larval zebrafish microbiota are well-characterized, and germ-free and gnotobiotic models are available (9).

The zebrafish zygote contains a large yolk cell which is absorbed over the first 5 days of life and supplies the developing embryo with nutrients. The yolk consists of a lipid and protein rich core with a cellular syncytium at its periphery, called the yolk syncytial layer (YSL). The YSL exports amino acids, hydrolyzes complex lipids to release fatty acids, and synthesizes lipoproteins, which export lipid to the developing embryo until it is able to feed independently (10). The intestine of the larval zebrafish is open at both ends and ready to absorb exogenous food at 5 dpf, though the non-enterocyte secretory cell populations do not differentiate until later larval stages (11). Once the intestinal tract is open, the gut microbiota are acquired from the media. At this time, colonization occurs essentially immediately and is maintained throughout life with the main source of variation in bacterial community composition being changes in diet (12).

Both the embryonic and larval zebrafish are valuable models of lipid uptake and trafficking, respectively, from the yolk cell and the diet. This review encompasses the roles of lipid remodeling, lipoproteins, intestinal lipid transport proteins, and the gut microbiota in lipid processing during zebrafish development.

## Yolk Lipid Uptake in the Embryonic and Larval Zebrafish

### Lipoproteins Transport Yolk Lipids to the Body of the Developing Zebrafish Embryo

**Figure 1:**
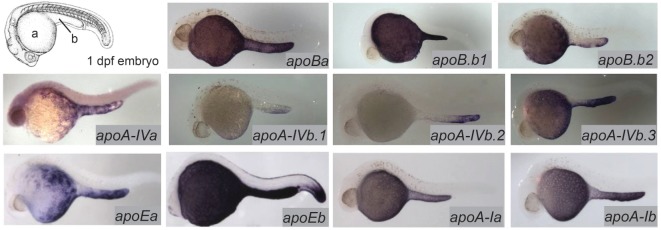
Zebrafish apolipoprotein genes are expressed in the yolk syncytial layer (YSL). The developing zebrafish embryo gradually absorbs lipids from its yolk (a), which is surrounded by the YSL. At 1–5 dpf, the yolk ball is lengthened along the tail of the embryo forming the yolk extension (b). *In situ* hybridization reveals expression of all 11 zebrafish apolipoprotein genes in the *apoB, apoA-IV, apoE*, and *apoA-I* families in the YSL at 1 day post-fertilization. Adapted and reprinted from Miyares et al. (18), and Otis et al. (13), under a CC-BY license.

The majority of the mass of a zebrafish zygote consists of the yolk, a lipid-rich structure that is gradually depleted by transport of its contents to the embryo as it develops into a free-feeding larva. Yolk lipids are packaged into lipoproteins in the YSL before being exported to the body of the developing zebrafish. Lipoproteins are lipid-transporting structures consisting of a neutral lipid interior bounded by a phospholipid (PL) and cholesterol monolayer, carrying one or more apolipoproteins. Apolipoproteins mediate interactions among lipoproteins, cellular receptors, and lipid-processing enzymes. The zebrafish genome contains analogs of every major human apolipoprotein, but there are some differences in patterns of expression and function. Due to the teleost genome duplication, zebrafish have multiple paralogs of each apolipoprotein gene. Human lipid metabolism genes with corresponding zebrafish paralogs discussed in this review are summarized in Table 1. There are 11 apolipoprotein genes in the *apoB, apoA-IV, apoE*, and *apoA-I* families, and all are expressed in the YSL (13) (Figure 1). Whole-mount *in situ* hybridization reveals that expression of some apolipoprotein genes is localized to subregions of the YSL, suggesting a previously uncharacterized compartmentalization of this structure. For example, mRNA encoding *apoA-IV* appears to be specific to the yolk extension at earlier stages (though different paralogs in this family are concentrated here at different points in development), while members of the other apolipoprotein families are expressed more evenly throughout the YSL (13). The significance of these potential YSL subdomains has yet to be described, but it is possible that there is a relationship to the regionalization of the developing intestine.

**Table 1:** Zebrafish express multiple paralogs of human lipid metabolism genes.

Product class	Human gene	Zebrafish gene (paralogs)	Expression in digestive tissues
Apolipoproteins	*apoA-I*	*apoA-Ia*	Yolk syncytial layer (YSL), larval intestine
		*apoA-Ib*	YSL, larval intestine, and liver

	*apoA-II*	*apoA-II*	YSL (14), larval, and adult liver (15)

	*apoA-IV*	*apoA-IVa*	YSL(extension), larval intestine
		*apoA-IVb.1*	YSL(extension), larval intestine
		*apoA-IVb.2*	YSL(extension), larval intestine
		*apoA-IVb.3*	YSL, larval intestine and liver

	*apoB*	*apoBa*	YSL, larval liver
		*apoBb.1*	YSL, larval intestine and liver
		*apoBb.2*	YSL, larval liver

	*apoC1*	*apoC1*	Larval and adult liver (15)

	*apoC2*	*apoC2*	Larval and adult liver (15)

	*apoE*	*apoEa*	YSL(extension), larval intestine
		*apoEb*	YSL, larval intestine

Cholesterol transporter	*npc1l1*	*npc1l1*	Adult intestine and liver (16)

Fatty acid transporters	*fatp3/acsvl3*	*fatp3/slc27a3*	

	*fatp1/acsvl5*	*slc27a1a*	
		*slc27a1b*	

	*fatp2/acsvl1*	*slc27a2a*	Adult liver
		*slc27a2b*	

	*fatp4/acsvl4*	*slc27a4*	Anterior larval intestine

	*fatp6/acsvl2*	*slc27a6*	

	*cav1*	*cav1*	Basal border of enterocytes

	*cd36*	*cd36*	YSL, larval intestine (17)

Long-chain Acyl-CoA synthetases	*acsl1*	*acsl1a*	Adult liver and intestine
		*acsl1b*	YSL, larval gut

	*acsl2*	*acsl2*	Adult liver

	*acsl3*	*acsl3a*	Adult liver and intestine
		*acsl3b*	Adult liver and intestine

	*acsl4*	*acsl4a*	YSL, larval gut, adult gut
		*acsl4b*	YSL, adult liver and intestine

	*acsl5*	*acsl5*	YSL, adult liver and intestine

	*acsl6*	*acsl6*	Adult liver and intestine

Although the expression of apolipoprotein genes in the developing embryo and larva has been thoroughly characterized, the lipoprotein profile at these stages is less well defined. Most work on fish lipoproteins has focused on adults, likely due to the difficulty of obtaining adequate blood samples from larvae (19). Secretion of very low-density lipoprotein (VLDL) particles from the yolk has been demonstrated by electron microscopy (20, 21). The YSL also expresses *apoA-I* and *apoA-II*, which are found in HDL (high-density lipoprotein) particles and chylomicrons but not LDL (low-density lipoprotein) or VLDL (13, 14). ApoB, which is a component of chylomicrons, LDL, and VLDL, has a vital role in the export of yolk lipids. Microsomal triglyceride (TG) transfer protein (MTP) packages TGs into ApoB-containing lipoproteins. ApoB is degraded if it is not associated with lipid so, in the absence of MTP function, ApoB is not functional (22). In *mtp^−/−^* mutant zebrafish larvae, lipids are trapped in the yolk (characterized by retention of yolk volume, an increase in yolk opacity, and a reduction in neutral lipid in the body) and larvae do not survive beyond 5 days (23). Additionally, unlike their wild-type siblings, *mtp^−/−^* embryos retain fluorescent fatty acid injected into the yolk and do not export it or its fluorescent products to the circulation (18). The ability of *mtp^−/−^* larvae to grow and survive to 5 dpf suggests that some lipid must be transported out of the yolk in order for membranes to be synthesized, possibly through the synthesis of HDL-like particles that contain ApoA-I and do not require MTP for their assembly.

### Lipid Composition of the Embryo Changes over the Course of Yolk Absorption

According to a recently published developmental study of lipid composition performed by liquid chromatography-mass spectrometry (LC-MS), at the time of fertilization, embryo lipids are approximately 40% cholesterol, 35% PL, and 9% TG, with less abundant species, including mono- and di-glycerides, cholesterol esters (CE), ceramides, and lysophospholipids, making up the remainder (24). Over the first 5 days of life, a linear decrease in the molar amount of most lipid species is observed in the yolk with a corresponding increase in the embryonic/larval body. Some exceptions have been observed: TG in the body remains consistently low as it is depleted from the yolk, suggesting that yolk TGs are primarily broken down and either oxidized for energy or resynthesized into other lipid products. Interestingly, CE, the other “energy storage” lipid class, is exchanged evenly from the yolk to the body during this period of development with the total amount remaining the same (24). Cholesterol synthesis in animal cells is tightly controlled in response to the cholesterol content of membranes *via* regulation of HMG-CoA reductase expression, and esterification is a major mechanism by which excess cholesterol is neutralized (25). One possible reason that CE is not depleted during the lecithotrophic (yolk-feeding) period of development is that breaking down CE for fatty acid oxidation would result in an overabundance of cholesterol. Favoring glycerolipids as an early energy source, therefore, would be important for cholesterol homeostasis, while CE from the yolk could be repackaged into intracellular lipid droplets for later oxidation or storage in adipocytes. Free cholesterol in the yolk and the body decrease and increase, respectively, at the same apparent rate between 24 h and 5 days of development, but the cholesterol content of the body at 5 dpf is less than the initial amount in the yolk (24). It is likely that this portion of the cholesterol is directed to synthesis of steroid hormones and bile, though these compounds were not measured in this study.

Phospholipid dynamics in the developing embryo also appear to be more complex than simple yolk to body trafficking: while other PL classes seem to move gradually from the yolk to the body, phosphatidylcholine (PC) levels in the yolk increase over the first 24 h, then decrease over the next 4 days while remaining relatively constant in the body (24). Though the specific lipid composition of zebrafish embryonic lipoproteins has not been investigated, one possible explanation is that the initial increase in PC goes to building the outer monolayer on lipoproteins exported from the yolk. It is possible that when this lipoprotein-associated PC reaches the body, it is in excess and is either oxidized or remodeled.

Although Fraher and colleagues’ published analysis of their LC-MS data set was limited to discussion of developmental changes in lipid classes, quantitation of all individual lipid species was published as a supplement to the manuscript. These data provide an opportunity to examine the changes in individual lipid species that occur during the first 5 days of zebrafish development. For example, the major PL classes are defined by head group (e.g., PCs, phosphatidylethanolamines, phosphatidylserines, etc.), but each of these classes comprises thousands of different molecules with different types of fatty acid “tails.” Modern mass spectrometry technologies optimized for lipidomics can differentiate between individual lipid species at this level of resolution because they can precisely determine the mass to charge ratio (*m/z*) of each analyte in a mixture and because they employ a second step in which the molecules are fragmented and the subsequent *m/z* values of these fragments are also determined. Complex lipids such as PLs are identified using *m/z* values calculated from molecular formulas and expected fragmentation patterns, and are annotated in Fraher’s supplemental data and other lipidomics data sets as “Head Group (FA 1/FA 2).” The most abundant PL in animal cell membranes, for example, is PC with the saturated 16-carbon fatty acid palmitate and the monounsaturated 18-carbon fatty acid oleate and is annotated as PC(16:0/18:1). When the specific fatty acid composition of a complex lipid cannot be determined, only the total fatty acid carbon chain length and number of unsaturated carbon–carbon bonds is given [e.g., PC(34:1)].

When trends in the amounts of individual lipids in Fraher’s data set are examined, results suggest that changes in the PL profile are consistent with an increase in membrane PL in the larval body that is expected to occur with increasing growth. However, the trends in total amounts of PL present in the yolk and body are skewed by changes in individual PL species. Specifically, PC(18:2/20:4) is a major PL in the body at the start of development and shows a large decrease by 5 dpf. However, the expected major PC components of cell membranes including PC(34:1) and other PCs with total chain lengths in the low 30s increase over the course of larval development as expected. It is possible that longer-chain PLs predominate in lipoproteins but are a minor species in cell membranes, a model supported by a large increase in the amount of PC(18:2/20:4) in the yolk over the course of development (this species is the only PL in the yolk whose total molar amount increases over 1–5 days, though other PL species increase in the yolk in terms of percentage of total lipid). PLs containing the fatty acid arachidonic acid (20:4) are the precursor of eicosanoids, a class of signaling molecules with roles in regulating inflammation, vascular physiology, and stem cell activity (26, 27).

This finding suggests eicosanoids as an important area of interest in the ongoing characterization of yolk utilization in the zebrafish. Although the physiological implications of changes in individual lipids were not within the scope of this published work, the rich MS data set that was produced highlights the importance of examining behavior of individual lipids in studies of metabolism and transport.

### Complex Lipid Synthesis and Remodeling Occurs in the Embryonic and Larval Zebrafish Yolk

The embryonic and larval zebrafish yolk is metabolically active not just in lipid transport, but also in the synthesis and remodeling of complex lipids, as was demonstrated through the injection of radioactive and fluorescently labeled lipids into the larval yolk followed by thin layer chromatography (TLC) analysis of the products of these metabolic tracers (18). Fatty acids labeled with BODIPY-FL?(4,4-difluoro-5,7-dimethyl-4-bora-3a,4a-diaza-*s*-indacene; a green fluorescent small molecule tag) or radioactive fatty acids injected into the yolk of 3 dpf larval zebrafish were both metabolized into complex lipids including PL, CE, and TG and transported throughout the developing body. Furthermore, injection of radioactive oleate showed that the yolk synthesizes complex lipids at the earliest stages of development, as radioactive TG and PL products were found in embryos injected as early as 0.75 hours post-fertilization (hpf). While the rate of incorporation of radioactive oleate into each PL class was consistent in embryos and larvae aged 0.75–3 dpf, larvae injected at 3 dpf were the only group to synthesize labeled CE, and there was a large increase in the amount of radioactive TG at later stages as well (18). When BODIPY-C12 was injected into the yolk of 24 hpf zebrafish embryos and yolk and body lipids were analyzed separately by TLC 1–6 h post injection (hpi), fluorescent complex lipids including TG, CE, and several unidentified species were produced in the yolk at early time points. Some fluorescent complex lipids were detected in the body at 6 hpi (24). (It is not known whether fluorescent PL was synthesized in this experiment as the assay only detected nonpolar lipids.) Injection of fluorescent PL into the yolk at 24 hpf resulted in fluorescent diglyceride and unidentified complex lipid species in the yolk, but no identified products in the body up to 6 hpi (24). Taken together, this and other evidence shows that the yolk is metabolically active throughout development and can both break down and synthesize complex lipids (18, 24, 28) (Table 2).

**Table 2:** The larval zebrafish is a versatile model system for metabolic labeling of lipids.

Labeled lipid substrate	Developmental stage/delivery method	Assay	Reference
Radioactive FA	1 dpf/yolk injection	Thin layer chromatography (TLC)	Miyares et al. (18)

	3 dpf/yolk injection	TLC	Miyares et al. (18)

	6 dpf/feeding	HPLC	Quinlivan et al. (29)

Fluorescent FA	1 dpf/yolk injection	TLC	Fraher et al. (24)

	3 dpf/yolk injection	TLC	Miyares et al. (18)

	6 dpf/feeding	TLC	Carten et al. (28)
		HPLC	Quinlivan et al. (29)

Fluorescent PL	1 dpf/yolk injection	TLC	Fraher et al. (24)

Fluorescent CE	6 dpf/feeding	HPLC	Quinlivan et al. (29)

## Dietary Lipid Uptake in the Larval Zebrafish

### Digestion and Absorption of Dietary Complex Lipids

The larval zebrafish undergoes a switch from a lecithotrophic state to a free-feeding animal during its fifth day of development, so by the time its yolk supply is depleted it must be able to digest and absorb nutrients from exogenous food sources. The ability to precisely control timing of the first meal is an advantage of this model as processing of dietary lipids by enterocytes can be observed without interference from lipids absorbed from previous meals. Additionally, because the larva retains its transparency for several weeks after it becomes free-feeding, it is possible to perform live imaging experiments with either single meals or ongoing defined diets in the same system.

Most dietary lipid consumed by animals enters the intestine not in the form of free fatty acids, but in complex lipids. Dietary TGs, PLs, and CE must be broken down by intestinal lipases in the lumen before the components of these molecules can cross the enterocyte membrane. As the fatty acids in these molecules are all linked by ester bonds, the intestinal lipases secreted by the exocrine pancreas are versatile and process a wide range of dietary lipids so that they can be absorbed (30). Following lipolysis, dietary lipid products form micelles in the intestinal lumen, which are emulsified in this aqueous environment by bile. The composition of bile varies between species and there are significant differences between teleost fish and humans, but its function is conserved (31, 32).

### Enteroendocrine Cells in the Intestine Regulate Digestion and Are Influenced by the Microbiota

As they do in mammals, enteroendocrine cells in zebrafish secrete a wide range of hormones including serotonin, which influences motility and appetite, and cholecystokinin (CCK), which stimulates gall bladder contraction and release of digestive enzymes from the pancreas (33, 34). The zebrafish genome contains two CCK paralogs; *ccka* is expressed in the digestive system of adults (no data are available for larvae at this time) and both *ccka* and *cckb* are expressed in the brain starting at 24 hpf (35, 36). In mammals, CCK promotes lipid digestion by stimulating the gall bladder to secrete bile, but does not increase lipase activity (37, 38). Similarly, larval zebrafish treated with a CCK receptor antagonist show reduced protease activity while intestinal phospholipase activity is unaffected (30). Enteroendocrine cells expressing serotonin begin to appear in the larval zebrafish intestine at 5 dpf. They may be detected by immunohistochemistry for serotonin, and are distinguished from the enteric neurons (which also express serotonin) by their shape and location in the epithelium. By 8 dpf, 10–18 enteroendocrine cells per larva may be observed in the distal intestine (posterior to the swim bladder) (11). A notable difference is that the larval zebrafish intestine does not have crypts, where enteroendocrine cells would be located in mammals.

The intestinal microbiota is required for normal enteroendocrine cell development (11). In germ-free larval zebrafish, 0–6 enteroendocrine cells were observed at 8 dpf (the total number of cells in the distal intestinal epithelium did not vary between germ-free and conventional groups). Larvae raised germ-free until 5 dpf, and then colonized with the conventional microbiota, developed normal numbers of enteroendocrine cells, suggesting that the yet-unidentified signal from the microbiota that promotes enteroendocrine cell development is not required before 5 dpf. Higher gut motility was observed in zebrafish larvae raised germ-free, suggesting a possible connection to digestive problems (including irritable bowel disease) observed in humans when the gut microbiota is disrupted (11). The lower number of serotonin-positive cells could explain this physiological effect as serotonin regulates gut motility in humans (33).

### Lipid Transport into Enterocytes

Dietary lipids are imported from the intestinal lumen across the apical enterocyte membrane by several different mechanisms depending on their class. After complex lipids (including both glycerolipids and CE) are digested to yield fatty acids, monoglycerides, and/or lysophospholipids, these products may cross membranes by a variety of transport processes conserved among zebrafish and mammals.

Cholesterol is taken up by enterocytes by a mechanism that requires the Niemann-Pick C1-Like 1 (NPC1L1) transport protein (39, 40). This membrane-associated protein is located at the brush border of enterocytes and is translocated to an intracellular compartment when cells are exposed to cholesterol; current models postulate a clathrin-dependent endocytic mechanism in which NPC1L1 is internalized along with a cholesterol cargo, which then moves through endosomes to the endoplasmic reticulum where it can be packaged into membranes or used to synthesize cholesterol ester (41, 42). NPC1L1 is encoded in the zebrafish genome, and several lines with point mutations in this gene have been created through the Sanger Institute Zebrafish Mutation Project (43). Ezetimibe, an inhibitor of NPC1L1-mediated cholesterol absorption that is used to treat hypercholesterolemia in humans, also blocks dietary cholesterol absorption in larval zebrafish (44–46). This creates an opportunity to use the zebrafish model to study physiological effects of modulating metabolic availability of a single component of a mixed-lipid diet. Regulation of NPC1L1 activity remains largely uncharacterized, although there is evidence from studies in humans given statins (inhibitors of cholesterol synthesis) that NPC1L1 expression levels increase in response to low intracellular cholesterol levels, suggesting that there may be an unidentified genetic mechanism that regulates NPC1L1 expression that could counteract the effects of statins by upregulating import of dietary cholesterol (47).

**Figure 2:**
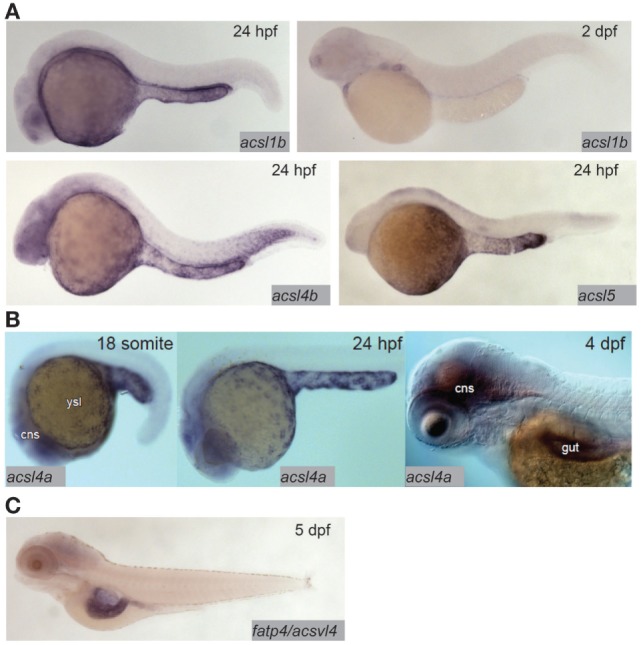
Acyl-CoA synthetases are expressed in the larval zebrafish yolk syncytial layer (YSL) and intestine. **(A)** *In situ* hybridization reveals expression of *acsl1b, acsl4b*, and *acsl5* in the YSL at 24 hpf, and *acsl1b* in the developing gut at 2 dpf. Adapted from Ref. (17). **(B)** acsl4a is expressed in the gut and central nervous system (cns) of the 4 dpf larval zebrafish, and in the YSL at 24 hpf and earlier. Reprinted from Ref. (52), Figure S1E in Supplementary Material, under a CC-BY license. **(C)** *fatp4*/*acsvl4* is expressed in the gut (especially the anterior bulb) of the 5 dpf larval zebrafish. Adapted from Ref. (53).

Fatty acid transfer proteins (FATPs) are a family of integral membrane proteins that facilitate transport of fatty acids into cells, including transport of dietary fatty acids into enterocytes. FATPs act in concert with acyl-coA synthetases (ACSs), which activate the newly imported fatty acids so that they are ready to form ester bonds and be incorporated into complex lipids (48, 49). There is evidence from mammalian and cell culture models that both the FATP and ACS families play roles in regulating preferential uptake of some dietary fatty acids over others, and in the partitioning of dietary fatty acids among complex lipids (48, 50). The zebrafish genome encodes 9 ACSL (ACSs specific to long-chain fatty acids, the type of fatty acid most abundant in animals including zebrafish) gene paralogs in six families. Expression of this class of genes is ubiquitous in adults, with proteins corresponding to seven of nine paralogs detectable by Western blot in most tissues including the gut (51). Expression of ACSL genes in the larva is more regionalized: in the *acsl1* family, *acsl1b* mRNA is detectable in the YSL and gut in early larval stages. The *acsl1a* paralog is not expressed in the YSL, and no expression data are available for early-gut development (17). Only *acsl1a* is expressed in the gut in adults (51). *Acsl4a* mRNA is present in both the YSL and the larval gut (52). Expression of *acsl4b* and *acsl5* is detectable in the YSL, but expression data are not available from larval stages after the gut has begun to develop (17) (Figure 2). Expression data are unavailable for the other *acsl* paralogs at any embryonic or larval stage, but what is known about expression of *acsl* genes in this model suggests potential division of function among paralogs similar to that suggested by regionalized apolipoprotein gene expression.

Compared with the ACSs, there is far less coverage of zebrafish FATPs in the current literature. As of now, no studies of FATP function in this model system have been published and only one genomic sequence is annotated as a FATP in the Ensembl database; FATP3/ACSVL3/SLC27A3 [with 7 paralogs, all annotated as members of solute carrier family 27 (*slc27*)]. The other six putative FATP paralogs are annotated as SLC27A1A and B {both with 65% protein sequence identity to human SLC27A1/FATP1/ACSVL5 [a mitochondrial long-chain FATP (54)], using the NCBI protein BLAST tool}, SLC27A2A (47% protein sequence identity to human SLC27A2/FATP2/ACSVL1), SLC27A2B (55% protein sequence identity to human SLC27A2/FATP2/ACSVL1), SLC27A4 (70% protein sequence identity to human SLC27A4/FATP4/ACSVL4), and SLC27A6 (57% protein sequence identity to human SLC27A6/FATP6/ACSVL2). The chromosomal locations of all of these putative *fatp* genes are conserved between the human and zebrafish genomes (syntenic analysis by ZFIN). Zebrafish SLC27A2A is expressed in the adult liver (55), and SLC27A4 is expressed in the anterior gut at 5 dpf (53) (Figure 2). No expression data are available for other adult organs, earlier larval stages, or the other putative FATPs at this time. However, as FATP4 is the primary fatty acid transporter on the apical brush border of human enterocytes, the similarity in expression between zebrafish and humans supports the larval zebrafish as a model in the investigation of FATP function in dietary fatty acid absorption (56).

The relative contributions of FATP4, other membrane-associated fatty acid-binding proteins, and passive diffusion to uptake of dietary fatty acids by enterocytes in larval zebrafish are not known. A recent review proposes a model in which the transmembrane receptor protein CD36, Caveolin 1 (Cav1), and FATP4 all act as fatty acid transporters at the enterocyte brush border, and in which passive diffusion of long-chain fatty acid salts across the enterocyte membrane plays a major role in adsorption (57). Larval zebrafish express CD36 and Cav1 in the intestine as well as FATP4 and, therefore, present an opportunity to apply live whole-animal imaging tools toward investigations of the roles of each of these proteins in dietary fatty acid processing (58, 59). [Cav1 is located on the basolateral membrane of enterocytes in zebrafish and not at the brush border and, therefore, is unlikely to participate directly in uptake of fatty acids from the intestinal lumen (59).] In sum, despite tight conservation of FATPs and other fatty acid transporters, and their intestinal expression throughout the vertebrates, their physiological role in the intestine remains unclear.

### The Intestinal Microbiota Influences Dietary Lipid Uptake

The bacterial population of the intestine also plays an important role in dietary lipid uptake and metabolism. Fermentation by the gut microbiota allows host animals to utilize dietary plant polysaccharides that would otherwise be indigestible by converting them to metabolizable short-chain fatty acids and monosaccharides (60). Multiple studies over the last decade have shown effects of changes in composition of the gut microbiota on adiposity, serum lipids, and tissue lipids in mammals (61–66). However, determining mechanisms by which bacteria may cause global changes in vertebrate host physiology has been difficult as the composition of the gut microbiota also changes in response to changes in diet (67, 68). The larval zebrafish model was recently used to investigate aspects of the relationship between gut bacteria and lipids involving processes other than short-chain fatty acid synthesis: when larvae raised germ-free were given a high-fat meal labeled with fluorescent fatty acids, less fluorescence accumulated in the intestinal epithelium when compared with conventionally raised larvae, showing that at least some members of the microbiota are necessary to promote uptake of dietary lipids. Monoassociated larvae (larvae raised germ-free and then inoculated with a single bacterial species) colonized with the Firmicutes strain *Exiguobacterium* sp. were used to demonstrate that this bacterial strain alone was sufficient to promote intestinal fatty acid uptake to a point where fluorescence could be observed in extra-intestinal tissues. Furthermore, experiments using conditioned media from this strain and two others also revealed significant increases in enterocyte lipid droplet number over untreated germ-free larvae, suggesting that a factor secreted by these species is involved in promoting dietary lipid uptake (69). The exact mechanism for this host–microbe relationship is currently uncharacterized, as is the evolutionary advantage of promoting host lipid uptake for these microbial species.

### Lipid Processing in Enterocytes for Storage and Export

Fatty acids taken up by enterocytes are repackaged into complex lipids at the endoplasmic reticulum and are subsequently stored in enterocyte lipid droplets or directed to lipoprotein synthesis for export. Lipid droplets are composed primarily of TGs and CE in the interior, and bounded by a PL monolayer with associated proteins such as perilipins (70). Though the mechanisms by which lipid droplets grow and shrink are well characterized, the regulation of lipid droplet size and number in various tissues is not as well understood, and most current research efforts focus on adipose and hepatic lipid droplets (71). As the intestine is not a site of long-term lipid storage in vertebrates including larval zebrafish, enterocyte lipid droplets are highly dynamic, temporary structures that respond with high sensitivity to the nutritive state of the animal. This property combined with the relative ease of live imaging in the larval zebrafish intestine compared with other animal models makes for an ideal system for the study of lipid droplet dynamics and regulation. When 5 dpf larvae are fed a high-fat/high-cholesterol meal of chicken egg yolk, both the average lipid droplet number per enterocyte and total area of the cell covered by lipid droplets increase significantly by 1 h post-feeding. Lipid droplet number peaks at 1 h and then gradually decreases, while total lipid droplet area is maintained up to 3 h following the meal, suggesting that smaller lipid droplets fuse as they mature (72). The gut microbiota also influence enterocyte lipid droplet number and size. Intestinal lipid droplets are both larger and more numerous in conventionally raised larvae after feeding than in germ-free larvae. Furthermore, conditioned media from a Firmicutes bacterial strain found to promote dietary fatty acid uptake and export to the liver was sufficient to increase enterocyte lipid droplet number but not the average lipid droplet size (69). These results have begun to reveal the diverse mechanisms by which different members of the gut microbiota influence lipid droplet dynamics and dietary lipid metabolism.

**Figure 3:**
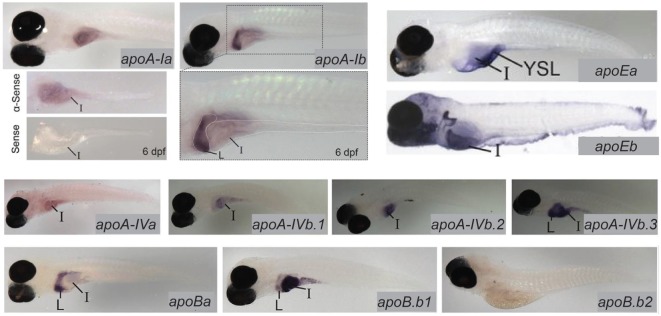
Zebrafish apolipoprotein genes are expressed in the larval digestive system. *In situ* hybridization reveals expression of 10 of the 11 zebrafish apolipoprotein genes in the *apoB, apoA-IV, apoE*, and *apoA-I* families in the liver and/or intestine of the 6 dpf larva. Dissected intestines probed for *apoA-Ia* are shown, and the gut of a larva probed for *apoA-1b* is magnified below the image of the whole larva. L, liver, I, intestine. Adapted and reprinted from Ref. (13), Figures 2–5, under a CC-BY license.

Lipoproteins are essential for the export of the products of dietary lipid from enterocytes into the circulation. Expression and function of apolipoproteins in the zebrafish is similar to that observed in mammals; at least one paralog from each of the ApoA-I, ApoB, ApoE, and ApoA-IV families is expressed in the larval zebrafish intestine (13). There is evidence that division of apolipoprotein function among organs is regulated by different mechanisms that achieve the same end in zebrafish and mammals: while different variants of ApoB are produced in the mammalian intestine and liver *via* RNA editing, larval zebrafish produce mRNA for the ApoB paralog b.1 in the intestine and liver and ApoBb.2 in the liver only. Similar compartmentalization of paralog expression between the liver and intestine is observed in the other apolipoprotein families as well (13) (Figure 3). Intestinal lipid accumulation in animals treated with an MTP inhibitor shows that as in the larval zebrafish yolk, availability of functional ApoB in necessary for normal rates of lipid export from the intestine, and that enterocyte lipid droplets are the destination of excess dietary fatty acids when export is slowed (73–75). The MTP inhibitor lomitapide is effective in larval zebrafish (72). It has also been observed that in mammals as the dietary fat content increases, chylomicron number reaches a plateau but average chylomicron size continues to increase, suggesting that apolipoprotein expression is the limiting factor in the rate of lipid export from the intestine (76).

### Total Lipid Biochemistry of the Larval Zebrafish Reveals Global Effects of Diet on Lipid Composition, and Facilitates Metabolic Labeling Studies

The larval zebrafish intestine is not only an excellent model for the study of lipid droplet and lipoprotein packaging, but also a site of differential channeling of dietary fatty acids depending on their chemical properties. The amenability of this model to biochemistry due to the ease of obtaining large numbers of embryos and larvae and performing lipid extractions from them, combined with the transparency of the larva, provides an opportunity unique among vertebrates to perform live imaging and metabolic labeling experiments in parallel using the same fluorescent lipid reagents (28, 77–79). Additionally, the whole-body lipid composition of the larval zebrafish is highly sensitive to changes in diet: the TG content of the 6 dpf larva increases 10-fold 24 h after a single high-fat meal (compared with a standard low-fat diet, and allowing time for the intestinal lumen to clear) (29). (In these experiments, the high-fat meal was chicken egg yolk; ~50% lipid dry weight, and the low-fat meal was SERA Micron larval growth food; 7% lipid. The lipid content of “standard chow” for zebrafish larvae is typically 5–15%.) Working at developmental stages before adipose tissue appears (~14 dpf) avoids signal to noise problems that may occur when the neutral lipid stored in adipose is included in the whole-body lipid profile. Also, at these early developmental stages examination of dietary lipid processing in the intestine can be isolated from potential regulatory influences from adipose tissue.

**Figure 4:**
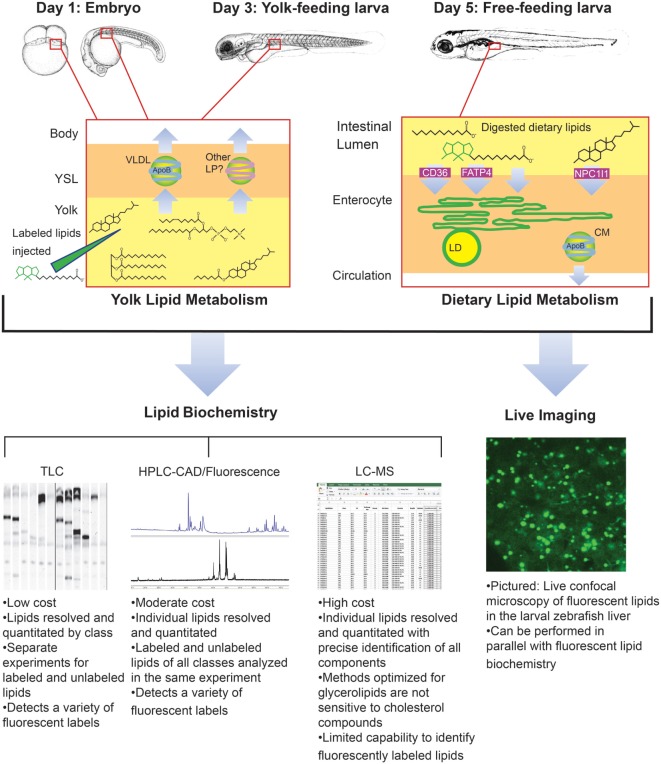
Metabolic labeling with fluorescent fatty acids is performed in the context of zebrafish development, yolk absorption, and dietary lipid metabolism. Fluorescent fatty acids (BODIPY-FL-C12 depicted) are trafficked and metabolized along with native yolk or dietary lipids when delivered to the developing zebrafish by yolk injection or feeding. LD, cytoplasmic lipid droplet, LP, lipoprotein, VLDL, very low-density lipoprotein. Embryo and larva illustrations adapted from Ref. (18).

Though it was beyond the scope of our recent metabolic labeling study (29), the biochemical techniques described therein could be applied to later-stage larvae in order to examine potential crosstalk between adipose tissue and the enterocytes that could influence dietary lipid partitioning. We have also developed methods for using fluorescent fatty acids as metabolic labels in the context of standard and lipid-enriched diets in larval zebrafish (Table 2). In addition to exploring the metabolic labeling potential of fluorescent lipids whose product profiles were not previously described, we have also applied HPLC with charged aerosol (total lipid detection) and fluorescence detection to obtain a greater depth of information than previous studies using fluorescent TLC (28). Initial findings indicate that the partitioning of saturated fluorescent fatty acids among complex lipid classes varies with carbon chain length, the total fat and cholesterol content of the diet, and the type of fluorescent tag (29). Metabolic labeling with fluorescent fatty acids in the context of lipid metabolism by the larval zebrafish is summarized in Figure 4.

Potential mechanisms regulating the rate of lipid export from the intestine beyond lipoprotein levels, the regulation and physiological effects of the size of enterocyte lipid droplets, and the channeling of newly absorbed dietary fatty acids into the different classes of complex lipids are currently largely uncharacterized. The optically clear and genetically tractable larval zebrafish model presents an ideal system in which to investigate these questions relating to energy homeostasis with a combined live imaging and biochemical approach.

## Author Contributions

VQ wrote the manuscript. SF edited the manuscript and provided guidance on the topic and scope.

## Conflict of Interest Statement

The authors declare that the research was conducted in the absence of any commercial or financial relationships that could be construed as a potential conflict of interest. The reviewer JM declared a past collaboration with one of the authors SF to the handling editor.
